# P-2026. Obeldesivir for the Treatment of COVID-19 in People with Risk Factors for Progression to Severe Disease: the BIRCH Study

**DOI:** 10.1093/ofid/ofae631.2182

**Published:** 2025-01-29

**Authors:** Anca Streinu-Cercel, Antonella Castagna, Shan-Chwen Chang, Yao-Shen Chen, Yiannis Koullias, Afsaneh Mozaffarian, Robert H Hyland, Rita Humeniuk, Luzelena Caro, Santosh Davies, Charlotte Hedskog, Shuguang Chen, Kim Etchevers, Nicole Behenna-Renton, Joe Llewellyn, Anu Osinusi, Frank Duff, Alejandro Barrat Hernández, Damien McNally, Jesus Abraham Simón Campos, Fabio E Leal, Leon Fouche, Juan M Gonzalez Del Castillo

**Affiliations:** "Prof. Dr. Matei Bals" National Institute for Infectious Diseases Carol Davila Medicine and Pharmacy University, Bucharest, Bucuresti, Romania; IRCCS San Raffaele Hospital and Vita-Salute San Raffaele University, Milano, Lombardia, Italy; National Taiwan University Hospital, Taipei, Taipei, Taiwan (Republic of China); Kaohsiung Veterans General Hospital, Kaohsiung, Kaohsiung; Gilead Sciences, Inc., Foster City, California; Gilead Sciences, Inc., Foster City, California; Gilead Sciences, Inc., Foster City, California; Gilead Sciences, Inc., Foster City, California; Gilead Sciences, Inc., Foster City, California; Gilead Sciences, Inc., Foster City, California; Gilead Sciences, Inc., Foster City, California; Gilead Sciences, Inc, Foster City, California; Gilead Sciences, Inc., Foster City, California; Gilead Sciences Europe Ltd, Uxbridge, England, United Kingdom; Gilead Sciences, Inc., Foster City, California; Gilead, Foster City, California; Gilead Sciences, Inc, Foster City, California; FAICIC Clinical Research, Veracruz, Veracruz-Llave, Mexico; Ormeau Clinical Trials, Belfast, Northern Ireland, United Kingdom; Köhler & Milstein Research/Hospital Agustín O’Horán, Mérida, Yucatan, Mexico; Brazilian National Cancer Institute; Faculdade de Medicina da Universidade Municipal de Sao Caetano do Sul, Rio de Janeiro; São Paulo, Rio de Janeiro, Brazil; Limpopo Clinical Research Initiative, Thabazimbi, Limpopo, South Africa; Emergency Department, Hospital Clínico Universitario San Carlos, Madrid, Madrid, Spain

## Abstract

**Background:**

COVID-19 remains a serious condition, as the risk of severe outcomes increases with age and comorbid conditions. Early antiviral therapy has been shown to prevent disease progression. Obeldesivir (ODV) is an oral, broad spectrum, nucleoside analog prodrug inhibitor of SARS-CoV-2 RNA-dependent RNA polymerase.

**Methods:**

BIRCH was a Phase 3, double-blind, placebo-controlled study in adults with risk factors for developing severe COVID-19 who were randomized 1:1 to ODV 350 mg or placebo twice daily for 5 days. The primary endpoint was COVID-19-related hospitalization or all-cause death through Day 29. Other endpoints included time to symptom alleviation and change in viral load and infectious titer. Safety assessments included adverse events (AEs) and laboratory abnormalities.

**Results:**

Study enrollment was stopped early, reflecting the evolving COVID-19 landscape with low rates of clinical events. Overall, 465 participants were randomized and received ≥1 dose. 56% were female, 4% were Black, 9% were American Indian or Alaska native, and 12% were Asian. Median age was 56 years; 29% were ≥65 years of age. 76% were randomized within 3 days of symptom onset, 42% had never been vaccinated, and 92% were SARS-CoV-2 seropositive. 90% were SARS-CoV-2 positive by nucleic acid amplification test at baseline for efficacy analyses. COVID-19-related hospitalization or all-cause death through Day 29 was reported in 0% for ODV (0/211 participants) and 0.5% for placebo (1/207 participants; *P* = 0.3161). Among the 162 participants who completed a symptom questionnaire, ODV showed a 2-day improvement in median time to symptom alleviation by Day 15 (ODV: 7.3 days; placebo: 9.3 days; *P* = 0.0859). ODV demonstrated greater reductions in viral load from baseline to Day 5 (–0.58 log_10_ copies/mL; *P* < 0.0001), and a greater proportion with negative infectious titer at Day 5 (ODV: 68/68 [100%]; placebo: 56/69 [81%]; *P* = 0.0001). The safety profiles of ODV and placebo were comparable, with similar rates of AEs, serious AEs, and AEs leading to discontinuation of study drug.

**Conclusion:**

ODV treatment resulted in greater decreases in viral load and infectious titer, and a trend in faster time to symptom alleviation. In this population of participants with risk factors for severe COVID-19, ODV was generally safe and well tolerated.

**Disclosures:**

Anca Streinu-Cercel, MD PhD Prof. Infectious Diseases, Gilead Sciences, Inc.: Grant/Research Support|Gilead Sciences, Inc.: Honoraria Antonella Castagna, MD, Bristol-Myers Squibb: Advisor/Consultant|Gilead Sciences, Inc.: Advisor/Consultant|Gilead Sciences, Inc.: Grant/Research Support|Gilead Sciences, Inc.: Honoraria|Janssen: Advisor/Consultant|Janssen: Grant/Research Support|Janssen: Honoraria|Merck Sharp & Dohme: Advisor/Consultant|Merck Sharp & Dohme: Grant/Research Support|Merck Sharp & Dohme: Honoraria|ViiV Healthcare: Advisor/Consultant|ViiV Healthcare: Grant/Research Support|ViiV Healthcare: Honoraria Yiannis Koullias, MD, Gilead Sciences, Inc.: Employee|Gilead Sciences, Inc.: Stocks/Bonds (Public Company) Afsaneh Mozaffarian, MS, Gilead Sciences, Inc.: Employee|Gilead Sciences, Inc.: Stocks/Bonds (Public Company) Robert H. Hyland, DPhil, Gilead Sciences, Inc.: Employee|Gilead Sciences, Inc.: Stocks/Bonds (Public Company) Rita Humeniuk, PhD, Gilead Sciences, Inc.: Former Employee|Gilead Sciences, Inc.: Stocks/Bonds (Public Company) Luzelena Caro, PhD, Gilead Sciences, Inc.: Employee|Gilead Sciences, Inc.: Stocks/Bonds (Public Company) Santosh Davies, MD, Gilead Sciences, Inc.: Employee|Gilead Sciences, Inc.: Stocks/Bonds (Public Company) Charlotte Hedskog, PhD, Gilead Sciences, Inc.: Employee|Gilead Sciences, Inc.: Stocks/Bonds (Public Company) Shuguang Chen, PhD, Gilead Sciences, Inc.: Employee|Gilead Sciences, Inc.: Stocks/Bonds (Public Company) Kim Etchevers, MS, Gilead Sciences, Inc.: Employee|Gilead Sciences, Inc.: Stocks/Bonds (Public Company) Nicole Behenna-Renton, BSc Hons, Gilead Science Europe Ltd: Employee|Gilead Science Europe Ltd: Stocks/Bonds (Public Company) Joe Llewellyn, PharmD, Gilead Sciences, Inc.: Employee|Gilead Sciences, Inc.: Stocks/Bonds (Public Company) Anu Osinusi, MD, Gilead Sciences, Inc.: Employee|Gilead Sciences, Inc.: Stocks/Bonds (Public Company) Frank Duff, MD, Gilead Sciences, Inc.: Employee|Gilead Sciences, Inc.: Stocks/Bonds (Public Company) Jesus Abraham Simón Campos, MD, AstraZeneca: Advisor/Consultant|AstraZeneca: Honoraria|Lilly: Advisor/Consultant|Lilly: Honoraria|Pfizer: Advisor/Consultant|Pfizer: Honoraria|Roche: Advisor/Consultant|Roche: Honoraria

